# Effects of music on post-stroke sleep disorders and treatment perspectives: review and narrative synthesis

**DOI:** 10.3389/fnhum.2025.1710535

**Published:** 2026-01-14

**Authors:** Shuangying Yang, Xue Yan, Ziwu Zhang, Wanning Gao, Tengyue Zhang, Weimin Zhang

**Affiliations:** 1 School of Rehabilitation Medicine, Changchun University of Chinese Medicine, Changchun, China; 2 Encephalopathy Department, The Third Affiliated Hospital of Changchun University of Chinese Medicine, Changchun, China; 3 Jingyue Fu'ao Community Health Service Station TCM Outpatient, Changchun, China

**Keywords:** mechanism, music therapy, sleep disorders, stroke, systematic review, treatment strategy

## Abstract

**Introduction:**

Sleep disorders represent the most prevalent psychiatric complication following stroke, seriously impacting patients' neurological recovery, functional prognosis, and quality of life. Music therapy, as a safe, cost-effective, and non-invasive intervention, is widely employed to ameliorate post-stroke sleep disorders. However, the precise mechanisms underlying music therapy's effects on post-stroke sleep disorders require further elucidation. This review aims to systematically examine the efficacy of music therapy for post-stroke sleep disorders and to elucidate the underlying physiological mechanisms through which music may improve sleep outcomes in this population.

**Methods:**

We conducted a systematic search across the Cochrane Library, PubMed, Embase, Web of Science, China National Knowledge Infrastructure (CNKI), Wanfang Data, and VIP, with a search time frame ending in August 2025. Quality assessment was performed using the Version 2 of the Cochrane risk-of-bias tool for randomized trials (RoB 2).

**Results:**

A total of 1,363 subjects from 14 original articles met the criteria for inclusion in the systematic review. Each study has shown that music intervention can effectively improve the sleep status in patients with post-stroke sleep disorders.

## 1 Introduction

Stroke represents the second leading cause of global mortality and the primary cause of adult disability among non-communicable diseases. Its incidence has risen significantly over recent decades, driven by evolving lifestyles, dietary patterns, and accelerating global population aging. Over the past 30 years, global stroke incidence has increased by approximately 70.0%, accompanied by a 43.0% rise in stroke-related mortality. The burden disproportionately affects low- and middle-income countries, which account for 83.3% of global cases. Notably high incidence rates are observed in the Solomon Islands (355/100,000), regions of sub-Saharan Africa, such as the Central African Republic (250/100,000), and several Southeast Asian nations, including Cambodia and Laos (200/100,000). In contrast, countries such as the United Arab Emirates (1/100,000) and the United States (2/100,000) report substantially lower rates (GBD 2021 Stroke Risk Factor Collaborators, 2024; Zhang et al., 2023). Post-stroke sleep disorders (PSSD) rank among the most prevalent complications, with a global prevalence ranging from 76.0 to 82.0%. Studies indicate acute sleep disorders affect up to 92.4% of ischemic stroke patients, and over half of stroke survivors experience sleep apnea, predominantly obstructive sleep apnea (Chen et al., 2024; Baillieul et al., 2022). PSSD poses significant clinical risks by impairing angiogenesis, axonal sprouting, and synaptogenesis. This exacerbates primary brain injury, ultimately hindering neurological recovery, diminishing rehabilitation efficacy, and elevating risks of disability, stroke recurrence, and mortality (Fengchun and Chen, 2023). Substantial evidence confirms a complex bidirectional relationship between stroke and sleep disorder (Pérez-Carbonell and Bashir, 2020; McDermott et al., 2018; Brunetti et al., 2022). Consequently, an in-depth investigation into PSSD treatment strategies is crucial for advancing stroke prevention and therapeutic outcomes.

Currently, Western pharmacological interventions and psychotherapy remain the primary clinical approaches for managing PSSD. Evidence indicates that medications such as eszopiclone, zolpidem, and mianserin demonstrate efficacy in improving sleep disturbances among stroke patients (Kim et al., 2017). However, these agents are frequently associated with significant adverse events. Research suggests that annual benzodiazepine use exceeding 4 grams or continuous use beyond 95 days elevates stroke incidence (Cai et al., 2021). Furthermore, frequent hypnotic use heightens fall risk during nocturnal bathroom use, particularly in elderly populations (Cai et al., 2021). The success of psychotherapeutic interventions, notably cognitive behavioral therapy (CBT), is substantially dependent on participants' cognitive status, and the long-term sustainability of treatment benefits remains uncertain (Belski et al., 2022). Alternative modalities, including transcranial direct current stimulation (tDCS), repetitive transcranial magnetic stimulation (rTMS), and acupuncture, offer advantages such as fewer side effects, broader applicability, and greater patient acceptance. Nevertheless, their protracted application can impose considerable financial burdens, especially within low- and middle-income countries and resource-limited families. Consequently, researchers are actively exploring novel therapeutic strategies. Among emerging options, music therapy (MT) has generated substantial interest as a safe, non-invasive, and cost-effective intervention.

The American Music Therapy Association (2025) defines MT as “The clinical and evidence-based use of music interventions to accomplish individualized goals within a therapeutic relationship by a credentialed professional who has completed an approved music therapy program, addressing patients' physical, emotional, cognitive, and social needs.” Fundamentally, MT is a systematic process of interventions designed to maintain, restore, and promote physical and psychological wellbeing. Substantial evidence supports its efficacy in improving sleep quality (Chen C. T. et al., 2021; Gou et al., 2024; Loewy, 2020). However, the specific effects and underlying mechanisms of MT for PSSD remain incompletely understood. Critical unresolved questions include: identifying optimal music types for PSSD; determining effective treatment protocols (encompassing duration, frequency, session number, and delivery methods); establishing validated efficacy evaluation tools; assessing whether MT offers greater benefits as an adjunctive therapy; and evaluating how patient baseline characteristics—including time since stroke onset, stroke subtype, comorbidities, and pre-existing conditions—impact intervention outcomes and necessitate protocol personalization. These multifaceted research gaps have garnered significant global attention. While a growing body of literature explores MT for PSSD, systematic integration and synthesis of these findings are lacking. To address this need and provide a comprehensive, in-depth understanding of the field, this review systematically synthesizes randomized controlled trial (RCTs) on the effects of MT for PSSD published within the past decade, and integrates their potential mechanisms.

This review aims to synthesize current evidence and elucidate the mechanisms of action underlying MT for PSSD, critically evaluating therapeutic outcomes associated with distinct music modalities, treatment protocols, and efficacy assessment methodologies. Through systematic analysis of RCTs, this work seeks to advance evidence-based clinical applications of MT for PSSD while identifying critical gaps in current evidence. Additionally, we provide researchers and clinicians with a comprehensive critical appraisal of methodological approaches in extant studies, thereby recommending robust methodological frameworks for future investigations that address the complex neurorehabilitative needs of this population.

## 2 Methods

We prepared the present study according to the Preferred Reporting Items for Systematic Reviews and Meta-Analyses (PRISMA; Page et al., 2021).

### 2.1 Literature search

We conducted a systematic search of the Cochrane Library, PubMed, Embase, Web of Science, China National Knowledge Infrastructure (CNKI), Wanfang Data, and VIP, using computerized searches to identify RCTs of maladaptive Knowledge-Based therapy for patients with PSSD, with a search time frame until August 2025. We obtained the keywords from the MeSH (Medical Subject Headings).

### 2.2 Inclusion and exclusion criteria

Inclusion criteria: participants: patients with a clinical diagnosis of stroke; and the Pittsburgh sleep quality index (PSQI) score ≥7 points; intervention: using music therapy, at least once a day; comparison: standards of care (SOC) for PSSD or SOC developed by hospitals based on expert consensuses or clinical guidelines; design: all the included studies were RCTs. Exclusion criteria: duplicates, animal studies, case reports, conference abstracts, reviews, unavailable full texts, and studies including participants with other organic diseases as comorbidities.

### 2.3 Data extraction

We read the full texts of the eligible articles and extracted the following data: study design, country, sample size, age gender, intervention, and outcome measures.

### 2.4 Data analysis

We calculated effect sizes as the mean difference (MD) using means and standard deviations (SD) for continuous outcomes, and as the risk difference (RD) or risk ratio (RR) for dichotomous outcomes. For any outcome addressed by three or more RCTs with similar interventions, we performed a meta-analysis to derive a pooled estimate. All statistical analyses were implemented in Python (version 12).

### 2.5 Risk of bias assessment

We assessed the risk of bias using the Version 2 of the Cochrane risk-of-bias tool for randomized trials (RoB 2) for assessing the risk of bias in randomized trials (Sterne et al., 2019).

## 3 Results

### 3.1 Process and results of literature screening

**Figure 1:**
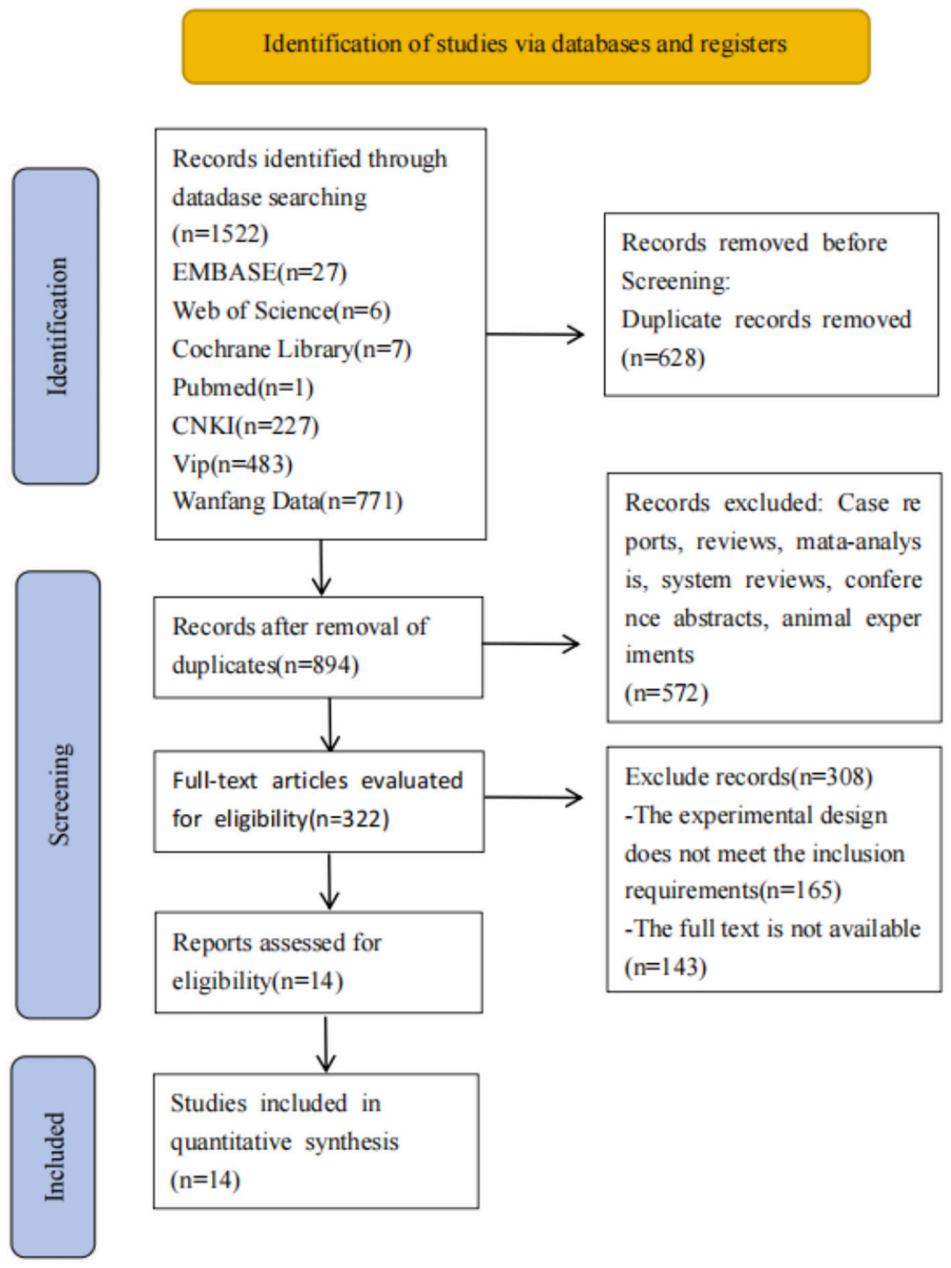
PRISlMA flow diagram of the study process.

We initially searched the database for 1,522 articles, and 628 duplicates were excluded. After the titles and abstracts, 572 reviews, systematic reviews, animal experiments, and other studies were excluded. After reading the full texts, 308 articles were excluded, and 14 were included (Figure 1).

### 3.2 Characteristics of included studies

A total of 14 articles involving 1,363 PSSD patients were included in the analysis. The music therapy program mainly used Five-Element Music, Western classical music or combined with drugs and acupuncture. The time of music intervention ranged from 2 weeks to several months. Most studies focused on the short-term therapeutic effects of music, with treatment durations typically around 30 min. Outcome indicators and efficacy evaluation mainly used PSQI, PSG, overall response rate, etc. The characteristics of the articles are detailed in Table 1.

**Table 1:** Characteristics of included articles.

**Included study**	**Country**	**Sample size**	**Age**	**Gender (M/F)**	**Intervention**				**Outcome measures**
					**E**	**C**	**Operators of music therapy**	**Basic features of music therapy**	
Ma, (2016)	China	E: 38 C: 38	E: 61.23 ± 7.13 C: 60.60 ± 7.19	E: 19/19 C: 20/18	Traditional Chinese folk music	Routine treatment	Not mentioned	40–60 dB 30 min/time 2 weeks	PSQI
Jia, (2021)	China	E: 25 C: 25	E: 57.12 ± 1.98 C: 56.77 ± 2.12	E: 12/13 C: 13/12	Five-Element Music Therapy	Oral estazolam tablets 1 mg/time 1 time/day 2 weeks	Not mentioned	40–60 dB 30 min/time 1 time/day 2 weeks	PSQI ORR AER
Chang et al., (2020)	China	E: 73 C: 73	E: 56.96 ± 10.52 C: 57.42 ± 11.23	E: 49/24 C: 47/26	Five-Element Music Therapy	Routine treatment	Not mentioned	25–35 dB 45 min/time 1 time/day 3 weeks	PSQI SRSS PSG
Yang et al., (2016)	China	E: 69 C: 68	E: 62.81 ± 6.99 C: 62.91 ± 7.76	E: 37/32 C: 42/26	Five-Element Music Therapy	Oral fluoxetine hydrochloride capsules 20 mg/time 1 time/day 2 weeks	Not mentioned	30 min/time 1 time/day 2 weeks	PSQI
Zhang et al., (2025)	China	E_1_: 25 E_2_: 24 C: 23	E_1_: 53.69 ± 7.02 E_2_: 54.02 ± 7.80 C: 54.2 ± 7.08	E_1_: 12/13 E_2_: 10/14 C: 15/8	E_1_: Five-Element Music Therapy E_2_: Western classical music	Routine treatment	Not mentioned	30 min/time 1 time/day 3 months	PSQI AIS PSG
Duan et al., (2018)	China	E: 46 C: 46	E: 63.4 ± 7.60 C: 63.6 ± 7.50	E: 25/21 C: 24/22	Group mode music therapy	Routine treatment + Sedative music	Professionally trained doctors, nurses, rehabilitation therapists	30 min/time 1 time/day 4 weeks	PSQI
Li, (2020)	China	E: 30 C: 30	E: 47.8 ± 10.2 C: 46.8 ± 10.6	E: 12/18 C: 13/17	Sedative music + Oral estazolam tablets 1 mg/time 1 time/day	Oral estazolam tablets 1 mg/time 1 time/day	Not mentioned	40–60 dB 30 min/time 1 time/day	PSQI ORR
Li et al., (2022)	China	E: 57 C: 57	E: 63.61 ± 6.43 C: 64.58 ± 6.73	E: 31/26 C: 30/27	Mozart's music Gregorian chant (high-low frequency conversion)	Routine treatment	Not mentioned	30 min/time 2 time/day 20 days	PSQI
Wang et al., 2019	China	E: 42 C: 43	E: 52.92 ± 15.49 C: 49.11 ± 17.47	E: 18/24 C: 26/17	Mozart's music Gregorian chant (high-low frequency conversion)	Mozart's music Gregorian chant	Neurologist, Rehabilitation specialist	30 min/time 2 time/day 30 days	PSQI
Huang and Deng, 2019	China	E: 57 C: 56	E: 64.52 ± 3.73 C: 59.72 ± 5.53	E: 21/19 C: 16/24	Five-Element Music Therapy + Doxepin hydrochloride tablets 6 mg/time, 1 time/day	Doxepin hydrochloride tablets 6 mg/time, 1 time/day	Not mentioned	40 min/time 1 time/day 3 weeks	PSQI ORR
Li et al., 2025	China	E: 35 C: 35	E: 66.69 ± 9.90 C: 65.31 ± 9.36	E: 21/14 C: 22/13	Five-Element Music Therapy + Leihuo moxibustion (GV20, HT7, ST36, BL15, BL20)	Leihuo moxibustion (GV20, HT7, ST36, BL15, BL20)	Not mentioned	30 min/time 2 time/day 14 days	PSQI ORR
Zhang et al., 2016	China	E: 52 C: 50	E: 64.50 ± 8.58 C: 62.30 ± 8.02	E: 25/27 C: 26/24	Five-Element Music Therapy + Auricular acupoint Sticking (Shenmen, AH_6a_, AT_1_, CO_15_, CO_12_, CO_10_)	Auricular acupoint Sticking (Shenmen, AH_6a_, AT_1_, CO_15_, CO_12_, CO_10_)	Not mentioned	30 min/time 1 time/day 4 weeks	PSQI ORR
Wang and Ding, 2013	China	E: 46 C: 46	E: 53.87 ± 7.60 C: 54.13 ± 7.792	E: 21/25 C: 19/27	Sedative music + Acupuncture (DU20, EX-HN3, HT7, EX-HN1, ST36, SP10, SP6, CV6), 30 min/time, 1 time/day, 3 weeks	Acupuncture (DU20, EX-HN3, HT7, EX-HN1, ST36, SP10, SP6, CV6), 30 min/time, 1 time/day, 3 weeks	Not mentioned	30 min/time 1 time/day 3 weeks	ORR SSI
Cai et al., 2015	China	E: 77 C: 77	E: 63.9 ± 10.4 C: 64.5 ± 12.6	E: 40/37 C: 43/34	Five-Element Music Therapy + Auricular acupoint Sticking (Shenmen, AH_6a_, CO_15_, CO_12_, CO_10_, AT_2, 3, 4)_	Auricular acupoint Sticking (Shenmen, AH_6a_, CO_15_, CO_12_, CO_10_, AT_2, 3, 4)_	Not mentioned	30 min/time 1 time/day 3 months	ORR

C, control; E, experimental; M, male; F, female; ORR, overall response rate; AER, adverse event rate; SRSS, self-rating scale of sleep; PSG, polysomnography; SSI, Spiegel Sleep Inventory; AIS, Athens Insomnia Scale.

### 3.3 Risk of bias assessment

Of the included studies, three do clearly state blinding methods, and two used non-randomized methods in the randomization process, thereby posing a high risk of bias. The other four high risk of bias arises mainly from incomplete data on outcome measures due to data loss. The risk of bias assessment of the included studies is shown in Table 2.

**Table 2:** Summery of the risk of bias.

**Study**	**Generation of random sequences**	**Allocation concealment**	**Blinding**	**Blinding of outcome evaluators**	**Incomplete data**	**Selective reporting**	**Other bias**
Ma, 2016	Unclear risk	Unclear risk	Unclear risk	Unclear risk	Low risk	Low risk	Unclear risk
Jia, 2021	Unclear risk	Unclear risk	Unclear risk	Unclear risk	Low risk	Low risk	Unclear risk
Chang et al., 2020	High risk	Unclear risk	Unclear risk	Unclear risk	Low risk	Low risk	Unclear risk
Yang et al., 2016	Unclear risk	Unclear risk	Unclear risk	Unclear risk	High risk	Low risk	Unclear risk
Zhang et al., 2025	Low risk	Unclear risk	Unclear risk	Unclear risk	High risk	Low risk	Unclear risk
Duan et al., 2018	Low risk	Unclear risk	Unclear risk	Unclear risk	Low risk	Low risk	Unclear risk
Li, 2020	Low risk	Unclear risk	Unclear risk	Unclear risk	Low risk	Low risk	Unclear risk
Li et al., 2022	Low risk	Unclear risk	Unclear risk	Unclear risk	High risk	Low risk	Unclear risk
Wang et al., 2019	Low risk	Unclear risk	Unclear risk	Unclear risk	High risk	Low risk	Unclear risk
Huang and Deng, 2019	High risk	Unclear risk	Unclear risk	Unclear risk	Low risk	Low risk	Unclear risk
Li et al., 2025	Low risk	Unclear risk	Unclear risk	Unclear risk	Low risk	Low risk	Unclear risk
Zhang et al., 2016	Low risk	Unclear risk	Unclear risk	Unclear risk	Low risk	Low risk	Unclear risk
Wang and Ding, 2013	Low risk	Unclear risk	Unclear risk	Unclear risk	Low risk	Low risk	Unclear risk
Cai et al., 2015	Low risk	Unclear risk	Unclear risk	Unclear risk	Low risk	Low risk	Unclear risk

### 3.4 Study results

We observed substantial heterogeneity in the interventions and outcome measures across the included studies. This precluded a pooled analysis of effect sizes. The results of each study—including means, SD, MD/RD/RR, and 95% Confidence Interval (95% CI)—are listed in Supplementary Appendix C. Below is a narrative synthesis of the evidence.

#### 3.4.1 Receptive music therapy

Ma (2016) compared the therapeutic efficacy of receptive music therapy with conventional care in a randomized controlled trial. Patients were allocated to either a receptive music therapy (RMT) group, which listened to 30 min of soothing music daily before bedtime, or a control group receiving standard rehabilitation training. Following a 2-week intervention period, the PSQI assessed sleep parameters in both groups pre- and post-treatment. Results demonstrated significant improvements in sleep onset latency, total sleep duration, and daytime functioning for the RMT group compared to controls. In addition, Duan et al. (2018) found that a structured program, that incorporated regular patient education and functional status assessments by a specialized medical team, produced better outcomes than music therapy alone.

Jia (2021) and Yang et al. (2016) compared the therapeutic efficacy of Five-Element Music Therapy with pharmacological treatment. The evaluation was conducted after the intervention using the PSQI and the overall response rate to assess treatment effects, along with the adverse event rate to monitor safety. Two weeks later, it was found that the music therapy group demonstrated superior efficacy and a more favorable safety profile than the drug-treated group. Chang et al. (2020) administered a 3-week Five-Element Music Therapy intervention to patients with PSSD and evaluated its effects relative to routine care using the PSQI, SRSS, and PSG. The results indicated that after 3 weeks of music therapy, significant improvements were observed in sleep quality, total sleep duration, and sleep efficiency. In a separate study, Zhang et al. (2025) compared the therapeutic outcomes of Five-Element Music, Western classical music, and routine care. Both types of music were found to improve sleep quality in PSSD patients; however, Five-Element Music demonstrated a more pronounced therapeutic effect. The authors suggest that the better efficacy of Five-Element Music Therapy may be partially explained by greater cultural affinity among Chinese participants, potentially enhancing its therapeutic acceptance and effects compared to Western musical forms.

Li et al. (2022) and Wang et al. (2019) investigated the effects of high-low frequency switching auditory-motor training (HLF-AMT) in patients with PSSD. HLF-AMT, a therapeutic approach developed by a French musicologist to enhance auditory processing skills, utilizes processed music to stimulate the cerebral cortex. This cortical activation mobilizes adjacent parietal musculature, modulating the ear-nervous system connection. The intervention durations were 20 days (Li et al., 2022) and 30 days (Wang et al., 2019). Sleep quality and the sleep-wake cycle were evaluated pre- and post-intervention using the PSQI and PSG. Both studies reported that the HLF-AMT groups exhibited significantly better outcomes than pre-intervention baselines and control groups across multiple metrics, including PSQI total score, specific sleep condition sub-scores, sleep latency, and total sleep time. The authors propose that processed audio effectively stimulates the vestibular and cochlear systems, thereby facilitating auditory-motor integration in the cortex for and enhancing sound processing. Concurrent stimulation of periauricular musculature may further activate the central nervous system, creating a more favorable physiological milieu for sleep.

However, the absence of reported follow-up data in these studies precludes conclusions regarding the long-term efficacy of RMT for PSSD.

#### 3.4.2 Active music therapy

Direct evidence regarding the application of active music therapy (AMT) for PSSD is currently lacking. However, studies confirm that psychological factors—including depression, anxiety, and diminished self-esteem—are key contributors to sleep disturbance development in stroke survivors (Yu et al., 2024). Moore (2013) posits that active engagement in MT, such as singing or improvisation, may positively influence mood regulation and activate cognitive control regions (e.g., the prefrontal, cortex and anterior cingulate cortex). Raglio et al. (2017) specifically demonstrated AMT's efficacy in improving psychological states, particularly reducing depression, in acute-phase stroke patients. Furthermore, their clinical research documented that patient engagement in instrumental playing beneficially impacted the functional recovery of impaired limbs; results confirmed significant improvement in hand and upper limb function among treatment participants. Thus, beyond ameliorating psychological distress, AMT stimulates motor function and promotes motor system neuroplasticity. This effect is likely mediated through the engagement of cortico-motor areas, auditory cortices, and integrated auditory-sensory-motor circuits.

Chronic insomnia and nocturnal awakenings in post-stroke patients may be associated with concerns regarding disease progression and residual motor deficits; addressing these concerns through AMT may thereby contribute to the alleviation of such sleep disturbances. Supporting this, Rushing et al. (2022) reported significant emotional state enhancement in stroke patients undergoing AMT, facilitated by patient-selected music during active participation. This active involvement fosters greater therapeutic engagement and positive treatment experiences. Therefore, AMT represents a promising therapeutic approach for addressing PSSD.

#### 3.4.3 Music combined with other therapeutic methods

Beyond standalone approaches, recent research has explored synergistic effects by integrating music therapy with complementary interventions. Li (2020) demonstrated superior sleep quality improvement when combining music therapy with eszopiclone vs. pharmacotherapy alone. Similarly, Huang and Deng (2019) applied Five-Element music therapy alongside doxepin for PSSD patients. Assessments using PSQI and ORR after 3 weeks revealed significantly better sleep quality and overall treatment outcomes in the combination group vs. monotherapy. These findings indicate that music therapy enhances conventional pharmacotherapy for PSSD, suggesting that there may be a synergistic effect in the mechanism: while hypnotic agents rapidly reduce cortical excitability to shorten sleep latency, music therapy potentiates physiological relaxation and consolidates therapeutic gains. Consequently, this integrated approach may facilitate dosage reduction, thereby minimizing drug dependence and adverse effects while improving efficacy.

Traditional Chinese medicine (TCM) leverages holistic principles and evidence-based modalities—including herbal medicine, dietary therapy, acupuncture, moxibustion, and tuina—to regulate qi-blood flow and yin-yang balance, offering favorable safety and acceptability profiles. Wang and Ding (2013) reported significantly higher efficacy rates for stroke insomnia patients receiving combined acupuncture and music therapy vs. acupuncture alone after 2 weeks. Similarly, Zhang et al. (2016) and Cai et al. (2015) demonstrated enhanced outcomes using music with auricular acupoint therapy vs. monotherapy. In addition, music combined with Leihuo moxibustion is also superior to simple Leihuo moxibustion in improving patients' sleep quality and depression (Li et al., 2025). Although music-TCM integration effectively ameliorates PSSD symptoms, further high-quality studies are warranted to validate these synergistic effects.

## 4 Potential physiological mechanisms of music therapy for post-stroke sleep disorders

A review of 14 studies, found that music-based intervention effectively alleviate symptoms associated with PSSD, suggesting their potential role in promoting neurofunctional recovery. The following section will elaborate on the underlying therapeutic mechanisms of music for PSSD. Although a comprehensive neurophysiological model of music therapy's mechanisms in PSSD remains to be fully elucidated, current evidence reveals multimodal physiological pathways. Crucially, auditory rhythmic entrainment synchronizes neural oscillations, modulating central nervous system activity and intrinsic physiological rhythms. Clinical studies demonstrate that music influences limbic system activation, subsequently regulating serotonin (5-HT) and dopamine (DA) neurotransmission. Concurrently, it modulates cerebral hemodynamics through neurovascular coupling, improving cerebral perfusion in sleep-regulatory networks. Preclinical models further indicate that music intervention downregulates pro-inflammatory cytokines, including IL-6, IL-1β, TNF-α, and CRP, thereby attenuating neuroinflammatory damage to sleep-regulatory centers following stroke. Additionally, the Five-Element theoretical framework of TCM music therapy offers a complementary paradigm for PSSD management, though its precise neuroanatomical substrates require empirical validation. Given these multidimensional mechanisms—encompassing neuromodulator, hemodynamic, and immunoregulatory pathways—this review systematically examines their potential integration as the neurobiological foundation for music-based interventions in PSSD, ultimately the development of evidence-based clinical protocols.

### 4.1 Rhythmic entrainment phenomenon

Modern medical research has established that MT induces rhythmic entrainment within the human body. This phenomenon involves the synchronization of internal physiological rhythms—such as respiration, heart rate, and neural oscillations—with external periodic rhythmic stimuli, including musical rhythms, mechanical oscillations, or photic stimulation (Varlet et al., 2015). The core neurophysiological mechanisms underpinning entrainment involve the oscillatory coupling within the nervous system, Central Pattern Generators (CPGs), and dynamic modulation of the autonomic nervous system. Auditory processing transmits music to the brain, where it is encoded as a temporal signal via neuronal phase-locking along the thalamocortical pathway. When perceived musical rhythms approximate intrinsic biological rhythms, the cerebral cortex initiates synchronization of respiratory, cardiac, and electrocortical activities through neural oscillations or forced entrainment. Alternatively, modulation may occur via direct projections through the auditory-brainstem pathway to respiratory and cardiovascular centers within the brainstem reticular formation. Furthermore, predictive coding enables anticipatory regulation of these centers and phase alignment of CPG output via cerebellothalamic pathways, establishing closed-loop feedback (Large and Snyder, 2009; Patel and Iversen, 2014; Nozaradan et al., 2011; Trost et al., 2012).

Empirical studies by Bernardi et al. (2006) and Thoma et al. (2013) demonstrate that low-frequency rhythmic music enhances parasympathetic tone, increases heart rate variability (HRV), and reduces blood pressure, heart rate, and respiratory rate—physiological markers associated with autonomic relaxation. Czepiel et al. (2021) observed significant synchronization of respiratory rate, heart rate, and electrodermal activity across 132 subjects exposed to classical music, noting enhanced entrainment efficacy with stable tempi and high listener familiarity. MT also modulates cortical activity, increasing alpha wave power—an electrophysiological correlate of physical and emotional relaxation (Daly et al., 2014). Critically, stroke disrupts core circadian gene expression, impairing sleep-wake cyclicity and blunting the nocturnal blood pressure dip (typically 10%−20%), which correlates with stroke severity (Lu et al., 2023; Zhang et al., 2022). The rhythmic entrainment generated by tempo-specific music may therefore represent a therapeutic mechanism for PSSD by facilitating the restoration of normative nocturnal blood pressure profiles. Collectively, these findings indicate that MT promotes sleep initiation by entraining respiratory rate, HRV, and brainwave activity.

### 4.2 Modulation of neurotransmitter systems

Beyond rhythmic entrainment, MT may ameliorate PSSD by modulating monoamine neurotransmitter systems, particularly 5-HT and DA. These neurotransmitters critically regulate mood, cognition (Jenkins et al., 2016), sleep architecture (Xia et al., 2024), and respiratory control (Aung et al., 2024). Serotonergic synthesis begins with tryptophan hydroxylase-mediated conversion to 5-hydroxytryptophan, followed by aromatic amino acid decarboxylase-dependent decarboxylation to 5-HT. This primarily occurs in the brainstem nucleus tractus solitarius (Sharp and Barnes, 2020). Of the seven receptor families and fourteen 5-HT receptor subtypes identified, 5-HT1A, 5-HT2A/2C, and 5-HT7 receptors are particularly implicated in sleep-wake regulation (Hoyer et al., 2002). Post-stroke reductions in 5-HT secretion—especially following lesions affecting serotonergic neurons—contribute to sleep fragmentation and PSSD pathogenesis (Chen et al., 2024). Supporting this, a randomized controlled trial demonstrated that low-frequency acupoint electrical stimulation elevated serum 5-HT levels and alleviated post-stroke insomnia (Tang et al., 2015). Furthermore, 5-HT dysregulation is associated with the development of obstructive sleep apnea, as evidenced by an inverse correlation between serum 5-HT levels and OSA severity scores (Wieckiewicz et al., 2023).

Dopamine, predominantly synthesized in the midbrain ventral tegmental area and thalamus, regulates sleep-wake cycles through multiple mechanisms: DAergic neurons facilitate rapid arousal transitions, reduce non-rapid eye movement sleep (NREM)-to-wake latency, and increase total wake duration (Sulaman et al., 2023). DA also modulates circadian gene expression and melatonin metabolism (Hou et al., 2025). Thalamic or thalamocortical circuit strokes disrupt DA secretion and projection (Han et al., 2024), while post-injury microglial activation (releasing IL-6 and TNF-α) may suppress dopamine synthase expression (Tang et al., 2021). Aberrant DA dynamics strongly correlate with post-stroke insomnia and restless legs syndrome (Li and Sun, 2020; Ruppert et al., 2017).

MT exerts unique neuromodulator effects on limbic circuitry. Music-evoked emotional responses (particularly from sedative music) enhance nucleus accumbens activity via auditory cortex-limbic-brainstem pathways, strengthening functional connectivity within limbic networks. This promotes 5-HT synthesis and release, corroborated by animal studies showing MT restores striatal 5-HT levels in stroke models (Yuan and Wu, 2021). Simultaneously, MT potently activates mesolimbic dopaminergic reward pathways; music-induced pleasure increases DA release, which further reinforces nucleus accumbens-limbic connectivity (Koelsch, 2014; Bernardi et al., 2009; Martínez-Molina et al., 2016). Notably, DA also potentiates rhythmic entrainment, enhancing physiological synchronization with musical rhythms (Patel and Iversen, 2014).

Thus, MT-mediated enhancement of 5-HT and DA neurotransmission represents a plausible therapeutic mechanism against PSSD. Important caveats exist: both neurotransmitter systems exhibit complex receptor subtype distributions with distinct neuroanatomical localization and functions (Hasegawa et al., 2024). Existing studies primarily measure neurotransmitter levels rather than receptor-specific effects—a methodological limitation that may introduce bias. Future research should employ advanced techniques to conduct quantitative assessments of MT's effects on neurotransmitter receptors, such as receptor autoradiography, PET imaging with subtype-specific ligands, and *in vivo* microdialysis.

### 4.3 Regulation of cerebral blood flow

Beyond its influence on rhythmic entrainment and its influence on neurotransmitter secretion and release, music modulates cerebral blood flow (CBF) dynamics. This modulation provides further evidence supporting music's beneficial effects on PSSD. Extensive research documents CBF alterations during human sleep: slow-wave sleep shows a slight reduction in CBF, whereas rapid eye movement (REM) sleep shows higher CBF and metabolic levels than wakefulness (Fultz et al., 2019). One study specifically reported significantly increased capillary CBF during REM, contrasting with typical decreases during non-rapid eye movement NREM sleep (Kotajima et al., 2005). Clinical observational studies further reveal a positive correlation between sleep quality and CBF in the right frontal and insular cortices (Park et al., 2021). Conversely, chronic insomnia patients show significantly reduced CBF in multiple regions, including the bilateral dorsolateral superior frontal gyri, the right middle frontal gyrus, the right anterior and posterior orbitofrontal cortices, the right inferotemporal gyrus, and the left lingual gyrus. Similarly, patients with NREM sleep disorders exhibit decreased CBF in the parieto-occipital lobe (anterior cuneus), limbic gyrus, and cerebellar hemispheres (Hanyu et al., 2011).

Following cerebral ischemia, blood flow to affected tissues diminishes drastically or ceases entirely. While restoring perfusion is essential for treating ischemic injury, reperfusion itself can paradoxically exacerbate tissue damage, amplifying disease progression (Chen Y. et al., 2021). The neurovascular unit (NVU)—comprising neurons, glial cells, vascular cells, and the basement membrane matrix within the cerebral vasculature—maintains brain homeostasis and regulates CBF under physiological conditions (Wang et al., 2021). Critically, the NVU also protects damaged neural tissues. Post-ischemic injury compromises NVU integrity and microvascular function, impairing its protective and hemodynamic regulatory roles, thereby promoting PSSD pathogenesis (Zhang et al., 2019). Additionally, post-stroke activation of the Rho/ROCK pathway reduces endothelial nitric oxide, synthase phosphorylation, thereby diminishing NO production. This stimulates contraction of vascular endothelial cells, further reducing CBF. Middle cerebral artery occlusion animal models demonstrate elevated ROCK activity in ischemic zones; administration of ROCK inhibitors suppresses this activation and increases CBF (Pinosanu et al., 2024; Kimura et al., 2021). Based on the summary by Bartel and Mosabbir (2021), exposure to 30–100 Hz music vibrations elevates Syndecan-4 and VEGF levels, triggering the release of endothelial NO. This process modulates vascular tension and increases blood perfusion, thereby enhancing circulatory function. This regulates vascular tone and blood flow, promoting circulation. Furthermore, NO induces BDNF and GDNF, conferring neuroprotective and therapeutic effects (Bartel and Mosabbir, 2021).

Music also influences cerebral hemodynamics via autonomic balance and respiratory modulation (Bernardi et al., 2006, 2009). Slow-tempo music enhances parasympathetic activity and reduces CBF, whereas fast-tempo music stimulates sympathetic nerves, increasing middle cerebral artery flow velocity. Music indirectly regulates CBF by modulating respiratory patterns and inducing cerebral vasomotion. Significantly, Bernardi et al., based on observations of 24 participants, found that the most significant relaxation effects often occurred during the silent intervals between musical phrases (Bernardi et al., 2006). Moreover, animal experiments and clinical trials demonstrated that 40 Hz gamma-frequency music selectively activates auditory pathways spanning from the brainstem to the cerebellum, increasing regional CBF in the contralateral auditory cortex, the dominant superior temporal gyrus, the ipsilateral postcentral gyrus, and the inferior temporal cortex (Chen et al., 2022). Consequently, personalized music therapy integrated with PSG represents a feasible strategy to enhance sleep quality in stroke patients. This could involve using slow music during SWS and NREM to decrease CBF, fast music during REM to increase CBF, and incorporating alternating tempos with silent periods to promote relaxation. Notably, individual patient characteristics require careful consideration; for instance, prolonged exposure to fast-tempo music should be avoided in the acute phase to prevent reperfusion injury potentially triggered by excessive CBF elevation and associated hypertension.

### 4.4 Reducing inflammation

MT may further ameliorate PSSD through immunomodulatory effects (Rebecchini, 2021). Post-stroke neuroinflammation, driven by cerebral hypoperfusion, leukocyte activation, pro-inflammatory mediator release, and blood-brain barrier (BBB) disruption, elevates systemic IL-6, IL-1β, TNF-α, and CRP—a profile consistently replicated in clinical and preclinical studies (Ohashi et al., 2023; Li et al., 2021). These cytokines exacerbate neuronal injury and represent key pathophysiological triggers for PSSD, given the established correlation between inflammatory markers and sleep architecture disruption (Zielinski and Gibbons, 2022). Specifically: IL-6 prolongs NREM sleep while suppressing REM phases, impairing cellular repair; TNF-α dysregulates sleep-associated brain regions and elevates CRP (Timmons et al., 2021; Latorre et al., 2022); IL-1β induces neuroinflammation while perturbing growth hormone signaling and HPA axis function via NF-κB/NLRP3 pathways, thereby fragmenting sleep-wake cycles (Kaushal et al., 2012).

Notably, MT demonstrates significant anti-inflammatory properties across species. Clinically, AMT engagement modulates immune biomarkers more effectively than passive listening, reducing pro-inflammatory cytokines while attenuating HPA axis hyperactivity (Rebecchini, 2021; Kang et al., 2018). Gamma-frequency music (40 Hz) may potentiate these effects by entraining cortical oscillations in auditory-hippocampal-prefrontal circuits, suppressing corticotropin-releasing hormone release, and enhancing GABAergic inhibition. Preclinical models confirm that MT downregulates IL-6, IL-1β, TNF-α, and CRP at both protein and transcriptional levels (Fu et al., 2023; Nian et al., 2023; Wang et al., 2024). Proposed mechanisms include: HPA axis normalization with cortisol stabilization; increased neurotrophic support and anti-apoptotic activity; reduced oxidative stress in prefrontal-hippocampal-amygdalar circuits; and suppression of NF-κB-mediated inflammatory cascades.

Critically, environmental noise exposure counteracts the benefits of music therapy by inducing neuronal apoptosis, impairing immune organs, and triggering behavioral disturbances. These findings underscore the importance of controlling ambient noise during therapeutic music implementation to mitigate noise-induced sleep disruption in hospital settings (Loewy, 2020).

### 4.5 Chinese traditional medicine's understanding of music therapy

TCM offers a distinct perspective on MT. TCM's conceptualization of MT dates back to the Yellow Emperor's Classic of Internal Medicine. As elucidated in the Ling Shu, “There are five tones in heaven, corresponding to the five zang organs in humans; there are six pitches in heaven, corresponding to the six fu organs in humans.” Further, Su Wen specifies: “The Gong tone pertains to the spleen, being broad and harmonious; excessive contemplation injures the spleen. An exuberant Gong tone can regulate this anger. The Shang tone pertains to the lungs, being light and strong. The Jue tone pertains to the liver and is harmonious and direct. The Zhi tone pertains to the heart, being rushing and beautiful. The Yu tone pertains to the kidneys, being deep and profound.” These classical passages establish the correspondence between the five tones/six pitches and the five zang/six fu organs, as well as the principle of emotional regulation through music. Traditional Chinese MT, grounded in syndrome differentiation (Bian Zheng) and the theoretical framework of the Five Elements, including their relationships of generation, restriction, and transformation, is consequently termed Five Elements Music Therapy. According to TCM theory: the Jue tone governs the liver, the Zhi tone governs the heart, the Gong tone governs the spleen, the Shang tone governs the lungs, and the Yu tone governs the kidneys. Although the lesion in PSSD resides in the brain, its pathogenesis is closely linked to dysfunction of the heart, liver, and spleen organs. Impairment of any of the five zang organs can precipitate sleep disorders, necessitating accurate syndrome differentiation prior to treatment.

During the acute phase of stroke or early recovery, patients typically present with excess syndromes, commonly including liver Yang hyperactivity, phlegm-heat internal disturbance, and blood stasis obstruction. Conversely, in the late recovery phase or chronic stage, deficiency syndromes predominate, such as heart-spleen deficiency, heart-kidney non-interaction, or Yin deficiency with effulgent fire. Once the patient's syndrome pattern is diagnosed, corresponding musical modes can be selected for intervention. For an organ identified as deficient, music corresponding to its “mother” organ is applied for tonification. If an organ's function is excessive, music corresponding to its “child” organ is used for drainage. Patients with complex syndromes may receive multiple musical modes applied alternately or in combination.

Similarly, Jia (2021) and Chang et al. (2020) employed different musical selections guided by pattern differentiation. Jia evaluated treatment efficacy using the Pittsburgh PSQI score, total effective rate, and adverse reaction incidence, while Chang utilized PSQI, PSG, and the Self-Rating Scale of Sleep. Both studies demonstrated improvement in PSSD symptoms. Furthermore, Guo et al. (2022) investigated the effects and mechanisms of Five Elements Music Therapy combined with acupuncture for acute stroke sleep disorder patients, using alprazolam as a control. Therapeutic efficacy was assessed by measuring serum cortisol, norepinephrine, and 5-HT concentrations, revealing superior outcomes in the Five Elements group after 4 weeks. In summary, Five Elements Music Therapy offers the advantages of evidence-based pattern differentiation and personalized treatment selection, demonstrating highly individualized characteristics and showing promising therapeutic effects for PSSD. However, its precise mechanisms of action and long-term efficacy warrant further in-depth investigation.

## 5 Conclusions and prospects

Patients with PSSD experience a constellation of functional deficits alongside significant sleep disturbances. Critically, stroke-induced motor and cognitive impairments substantially compromise both the implementation of conventional sleep interventions and patient adherence, thereby limiting therapeutic efficacy. Concurrently, persistent sleep disorders exacerbate emotional distress and impair daytime alertness, while simultaneously diminishing patients' motivation and engagement in rehabilitation. This bidirectional relationship directly undermines treatment outcomes and impedes functional recovery. The comorbid burden of stroke and sleep disorder is considerably greater than the impact of either condition alone, resulting in amplified repercussions for patients, their families, and society. As the healthcare paradigm shifts from a traditional biomedical model toward a biopsychosocial approach, the limitations of conventional PSSD management become increasingly apparent. Consequently, there is an urgent need to develop safer, more effective, and readily acceptable novel therapeutic approaches.

**Figure 2:**
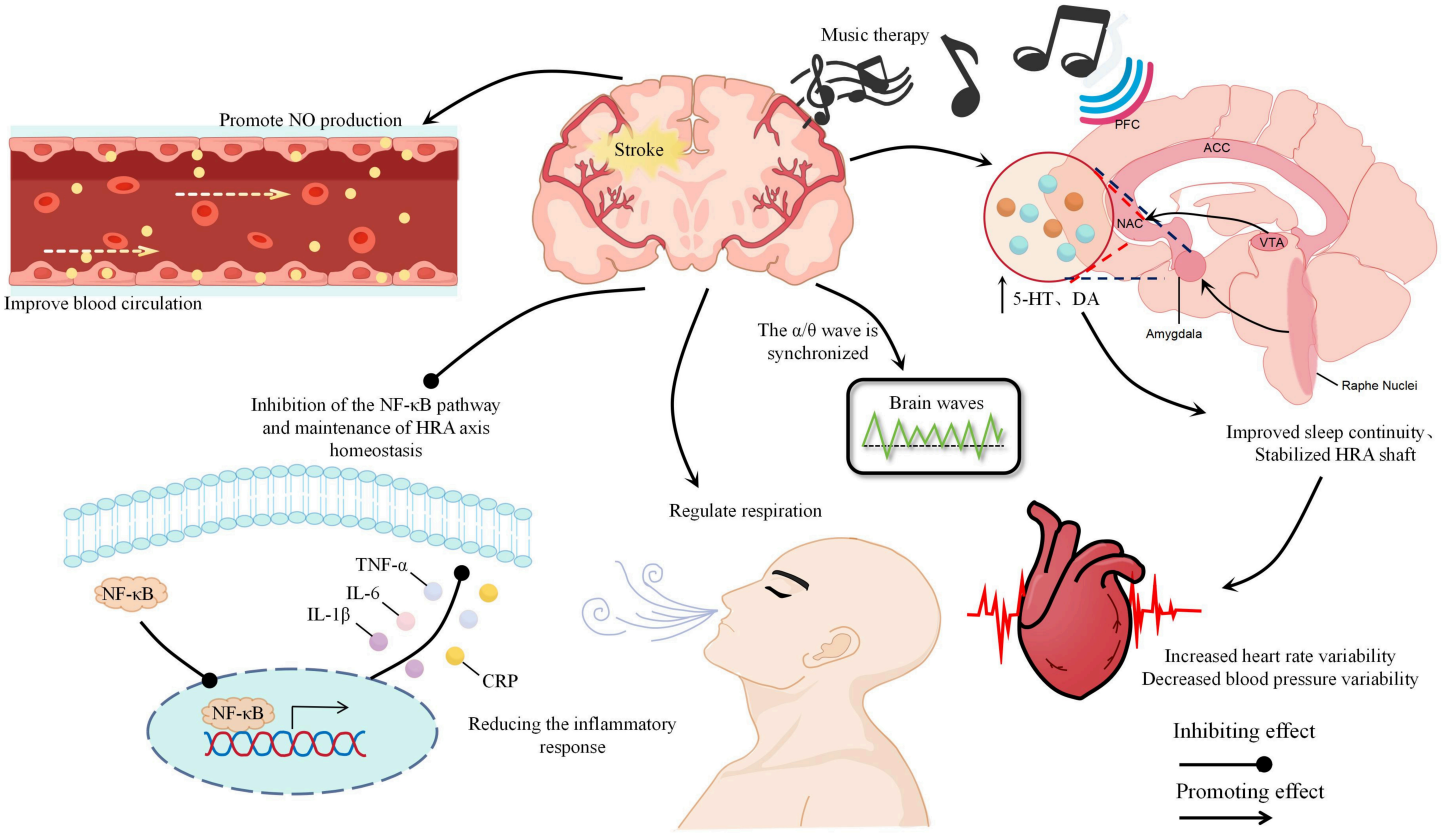
MT exerts therapeutic effects for PSSD through multiple pathways. Rhythmic entrainment helps normalize respiratory rhythm, HRV, and electroencephalographic activity to facilitate sleep initiation. Furthermore, MT enhances limbic system connectivity, increasing 5-HT and DA release to regulate the sleep-wake cycle. It also improves cerebrovascular perfusion by upregulating NO. Additionally, its anti-inflammatory action—mediated by suppressing NF-κB activation and rebalancing the HPA axis—reduces levels of IL-6, IL-1β, TNF-α, and CRP, thereby mitigating sleep center damage.

Substantial evidence indicates that MT exerts significant therapeutic effects on PSSD. This review elucidates key physiological mechanisms underpinning MT for PSSD (Figure 2), encompassing rhythmic entrainment, neurotransmitter modulation, cerebral hemodynamic regulation, anti-inflammatory actions, and the theoretical framework of the five elements within TCM. Rhythmic entrainment modulates intrinsic physiological oscillations, influencing respiratory rate, HRV, and electroencephalographic activity, thereby facilitating accelerated sleep onset. Post-stroke disruptions in 5-HT and DA secretion contribute to sleep pathology; MT counteracts this by activating the nucleus accumbens and limbic system, enhancing their functional connectivity, and promoting 5-HT and DA release, consequently ameliorating PSSD symptoms. Stroke-induced alterations in cerebral hemodynamics further compromise sleep. MT regulates cerebral blood flow and velocity through multiple pathways, including respiratory modulation, autonomic nervous system balance, and direct vascular effects. Additionally, stroke elevates systemic inflammatory markers. MT attenuates inflammation by suppressing NF-κB signaling, restoring HPA axis homeostasis, and acting via other mechanisms. In TCM theory, music is classified into five tunes corresponding to the heart, liver, spleen, lungs, and kidneys; dysfunction in any organ may precipitate sleep disorders, necessitating differentiation of symptom pattern for appropriate musical prescription. Regarding clinical application, receptive MT remains the predominant approach investigated for PSSD. While direct evidence for AMT in PSSD is limited, emerging studies suggest its potential benefits. Furthermore, combining MT with established therapies—such as pharmacotherapy and TCM—has been show to enhanc efficacy. Notably, MT also yields positive secondary outcomes, significantly improving depression, anxiety, motor function, and cognition in stroke patients. These improvements likely enhance patient engagement in rehabilitation, thereby optimizing overall recovery.

Despite the promising therapeutic potential of music for PSSD, several challenges impede its clinical implementation. First, the lack of standardized MT practical guidelines has lead to significant heterogeneity in treatment duration, intervention frequency, musical repertoire curation, auditory parameters, and therapeutic environments. Notably, existing ISO guidelines for MT lack specific applicability to stroke populations, compromising treatment fidelity and reproducibility in clinical applications. Second, while PSG remains the gold standard for objective sleep assessment, it demonstrates limitations in detecting specific sleep architecture disruptions (e.g., insomnia phenotypes) and exhibits poor concordance with subjective insomnia experiences. Additionally, its cost-prohibitive nature and concerns about inter-rater reliability warrant consideration (Frase et al., 2023). Third, although the PSQI reliably measures global sleep quality, it possesses inherent limitations in quantifying microarousals and sleep fragmentation, creating critical assessment gaps (Windmill et al., 2024).

Methodological constraints further complicate evidence interpretation. Many RCTs employ MT as an adjunctive therapy without dedicated MT comparator groups, thereby precluding definitive conclusions regarding MT's isolated efficacy for PSSD. Several studies also inadequately report exclusion criteria for post-stroke cognitive impairment and visual deficits, introducing potential confounding variables that undermine the validity of results. The efficacy and reproducibility of music therapy depend on professional delivery. However, only two studies clearly reported that certified therapists administered the intervention (Wang et al., 2019; Duan et al., 2018), a methodological gap that could bias the reported therapeutic effects. To strengthen future evidence, study protocols should explicitly require and describe the involvement of accredited therapists to mitigate this confounding factor.

Theoretical frameworks such as Five-Element Music Therapy emphasize pattern differentiation and individualized prescriptions. However, their application in the management of PSSD lacks robust mechanistic validation. To date, only one study (Zhang et al., 2025) has compared this approach with Western classical music, but the findings may have been confounded by cultural background and musical preference. Music is inherently shaped by cultural and situational factors, leading to distinct musical preferences across different populations. Research by Czepiel et al. (2021) also demonstrates that familiar music generates more beneficial physiological effects. Consequently, personalized treatment plans that account for cultural background and musical preference should be integrated into clinical practice to maximize therapeutic benefits. Although preliminary results from Zhang et al. (2016) and Jia (2021) indicate that music therapy is well tolerated, this conclusion is based on a limited number of reports, underscoring the need for more systematic safety monitoring in future investigations. Similarly, the absence of follow-up data in all reviewed studies consequently weakens the credibility of the reported outcomes. Future research should therefore incorporate structured follow-up assessments to verify the durability of therapeutic benefits. Finally, critical knowledge gaps persist regarding how stroke-specific factors (lesion location, time since onset, neurological deficit severity, and PSSD subtype) influence MT efficacy and whether these variables necessitate protocol personalization.

Future research should prioritize elucidating the role of music stimulation in modulating key pathological processes in PSSD and associated signaling pathways using standardized animal models. Clinically, multimodal assessment frameworks integrating sleep scales, PSG, biomarker analysis, and functional near-infrared spectroscopy for prefrontal oxygenation monitoring should be employed (Huang et al., 2021). Complementary neuroimaging techniques—particularly electroencephalography, electrooculography, and fNIRS—could collectively map dynamic alterations in interregional brain connectivity across sleep stages following music therapy. Synchronized neural activity recordings would further clarify the mechanisms underlying the coupling between specific acoustic parameters and neural oscillation patterns.

Therapeutic optimization requires analyzing: (1) differential effects of MT modalities; (2) stage-appropriate interventions (e.g., active MT for motor rehabilitation during recovery phases); and (3) evidence-based selection of rhythmic frequencies (e.g., accelerated rhythms to transiently augment cerebral blood flow in acute stages vs. slower tempi to stabilize perfusion during convalescence). Population stratification must account for comorbidities influencing stroke-sleep pathology interactions. Notably, post-stroke depression—a prevalent complication strongly associated with PSSD—exhibits high rates of acute-phase sleep disturbances (Cai et al., 2021). Given MT's established efficacy for PSD (Dayuan et al., 2022; Zhong et al., 2024), its potential indirect sleep benefits through mood modulation warrant investigation.

Methodological rigor requires stringent control of baseline confounders: systematic documentation of hypnotic medication use adequate washout periods to eliminate pharmacological interactions, and verification that inclusion criteria reflect patients' true clinical status. Studies by Roy-O'Reilly and McCullough (2018) have demonstrated that age and sex significantly influence both the incidence of ischemic stroke and subsequent functional recovery. Furthermore, post-stroke mechanisms—including neuroprotection, cell death pathways, and pharmacological responses—also exhibit distinct sex-related variations. Thus, it is imperative to incorporate sex and age-specific analyses in future MT research, particularly in studies examining its therapeutic effects and underlying neurophysiological mechanisms. Implementation safety requires real-time protocol adjustments based on therapeutic response monitoring. While MT exhibits favorable safety profiles, contingency planning for potential adverse events remains essential without compromising therapeutic observation. Adjunctive integration with respiratory biofeedback and guided imagery may further potentiate treatment efficacy.

Previous research has shown that post-stroke activation of Rab7a drives the degradation of selective tight junction proteins, thereby increasing BBB permeability. This facilitates an influx of peripheral inflammatory mediators that exacerbate neural tissue damage (Cottarelli et al., 2025). Notably, Sachdeva et al. (2022) have summarized that specific acoustic frequencies can modulate BBB permeability and alleviate neurovascular injury. We therefore hypothesize that the structured rhythmic elements of music may facilitate the normalization of BBB function during stroke recovery via frequency-dependent mechanisms. Elucidating how specific musical parameters—such as beat, rhythmic spectra, and resonant frequencies—influence BBB dynamics represents a promising frontier in understanding the therapeutic role of music therapy for PSSD. This proposed neuro-acoustic pathway warrants further experimental validation as a potential target for future interventions.

Collectively, the existing evidence indicates that MT effectively enhances sleep quality and mitigates the detrimental effects of sleep disturbances on daily functioning in patients with PSSD. However, a significant paucity of high-quality clinical research persists, necessitating further rigorously designed studies to address current methodological limitations. Future investigations should prioritize controlled trials with robust bias mitigation strategies to strengthen causal inference. Complementary mechanistic studies employing animal models are warranted to elucidate the underlying neurophysiological pathways and key therapeutic targets of MT in PSSD. Ultimately, this integrated research approach will enable the development of scientifically grounded, evidence-based clinical protocols for standardized MT implementation in PSSD management.

## 6 Limitations

To our knowledge, this review represents one of the most comprehensive syntheses to date regarding the clinical effects and potential mechanisms of music-based interventions. Nevertheless, several limitations should be acknowledged. First, all included studies were conducted in China, and the majority used interventions with distinctive TCM characteristics, which may limit the generalizability of the findings to other cultural or clinical settings. Second, considerable heterogeneity in study designs and outcome measures was observed across the included trials, owing to the diversity of intervention types, we could not combine their results in a meta-analysis. This is also the article's most obvious deficiency.
