# Supplementary material for: Effects of music on post-stroke sleep disorders and treatment perspectives: review and narrative synthesis
Source: Front Hum Neurosci. 2026 Jan 14;19:1710535. doi: 10.3389/fnhum.2025.1710535 (PMC12847286; doi:10.3389/fnhum.2025.1710535)
Supplement: Supplementary file 1 [file Data_Sheet_1.pdf]

Appendix a: Common database retrieval strategies

Database	Retrieval strategy
EMBASE	#1 'sleep disorder'/exp<br>#2 'atypical sleep':ti,ab,kw OR 'difficult sleeping':ti,ab,kw OR 'difficulties in sleeping':ti,ab,kw OR 'difficulty sleeping':ti,ab,kw OR 'disorder during sleep':ti,ab,kw OR 'disorder sleep':ti,ab,kw OR 'disorders during sleep':ti,ab,kw OR 'disorders sleep':ti,ab,kw OR 'disturbance during sleep':ti,ab,kw OR 'disturbance of sleep':ti,ab,kw OR 'disturbances during sleep':ti,ab,kw OR 'disturbances of sleep':ti,ab,kw OR 'problem during sleep':ti,ab,kw OR 'problem of sleep':ti,ab,kw OR 'problems during sleep':ti,ab,kw OR 'problems of sleep':ti,ab,kw OR 'sleep disorders':ti,ab,kw OR 'sleep disturbance':ti,ab,kw OR 'sleep disturbances':ti,ab,kw OR 'sleep interference':ti,ab,kw OR 'sleep perturbation':ti,ab,kw OR 'sleep perturbations':ti,ab,kw OR 'sleep problem':ti,ab,kw OR 'sleep problems':ti,ab,kw OR 'sleep related disease':ti,ab,kw OR 'sleep related disorder':ti,ab,kw OR 'sleep related problem':ti,ab,kw OR 'sleep wake disorder':ti,ab,kw OR 'sleep wake disorders':ti,ab,kw OR 'sleeping difficulties':ti,ab,kw OR 'sleeping difficulty':ti,ab,kw OR 'sleeping disorder':ti,ab,kw OR 'sleeping disorders':ti,ab,kw OR 'sleeping problem':ti,ab,kw OR 'sleeping problems':ti,ab,kw OR 'trouble sleeping':ti,ab,kw OR 'sleep disorder':ti,ab,kw<br>#3 'cerebrovascular accident'/exp<br>#4 'accident, cerebrovascular':ti,ab,kw OR 'acute cerebrovascular lesion':ti,ab,kw OR 'acute focal cerebral vasculopathy':ti,ab,kw OR 'acute stroke':ti,ab,kw OR 'apoplectic stroke':ti,ab,kw OR 'apoplexia':ti,ab,kw OR 'apoplexy':ti,ab,kw OR 'blood flow disturbance, brain':ti,ab,kw OR 'brain accident':ti,ab,kw OR 'brain attack':ti,ab,kw OR 'brain blood flow disturbance':ti,ab,kw OR 'brain insult':ti,ab,kw OR 'brain insultus':ti,ab,kw OR 'brain vascular accident':ti,ab,kw OR 'cerebral apoplexia':ti,ab,kw OR 'cerebral insult':ti,ab,kw OR 'cerebral stroke':ti,ab,kw OR 'cerebral vascular accident':ti,ab,kw OR 'cerebral vascular insufficiency':ti,ab,kw OR 'cerebro vascular accident':ti,ab,kw OR 'cerebrovascular arrest':ti,ab,kw OR 'cerebrovascular failure':ti,ab,kw OR 'cerebrovascular injury':ti,ab,kw OR 'cerebrovascular insufficiency':ti,ab,kw OR 'cerebrovascular insult':ti,ab,kw OR 'cerebrum vascular accident':ti,ab,kw OR 'cryptogenic stroke':ti,ab,kw OR 'cva':ti,ab,kw OR 'insultus cerebralis':ti,ab,kw OR 'ischaemic seizure':ti,ab,kw OR 'ischemic seizure':ti,ab,kw OR 'stroke':ti,ab,kw OR 'thrombotic stroke':ti,ab,kw OR 'cerebrovascular accident':ti,ab,kw<br>#5 'music therapy'/exp<br>#6 'melotherapy':ti,ab,kw OR 'therapy, music':ti,ab,kw OR 'music therapy':ti,ab,kw<br>(#1 OR #2) AND (#3 OR #4) AND (#5 OR #6)
Web of Science	#1 ((TS=(music therapy)) OR TS=(therapy,music)) OR TS=(music)<br>#2 ((((((((((((((((TS=(DIMS)) OR TS=(Disorders of Initiating and Maintaining Sleep)) OR TS=(Sleeplessness)) OR TS=(Insomnia Disorder)) OR TS=(Insomnia Disorders)) OR TS=(Insomnia)) OR TS=(Insomnias)) OR TS=(Chronic Insomnia)) OR TS=(Early Awakening)) OR TS=(Nonorganic Insomnia)) OR TS=(Primary Insomnia)) OR TS=(Psychophysiological Insomnia)) OR TS=(Rebound Insomnia)) OR TS=(Secondary Insomnia)) OR TS=(Sleep Initiation Dysfunction)) OR TS=(Dysfunction, Sleep Initiation)) OR TS=(Transient Insomnia)<br>#3 (((((((((((((((((((TS=(Strokes)) OR TS=(CVA (Cerebrovascular Accident))) OR TS=(Apoplexy)) OR TS=(Cerebrovascular Accident)) OR TS=(Cerebrovascular Stroke)) OR TS=(Vascular Accident, Brain)) OR TS=(Brain Vascular Accidents)) OR TS=(Cerebral Stroke)) OR TS=(Cerebrovascular Apoplexy)) OR TS=(Stroke, Cerebral)) OR TS=(Apoplexy, Cerebrovascular)) OR TS=(CVAs (Cerebrovascular Accident))) OR TS=(Cerebrovascular Accidents, Acute)) OR TS=(Cerebrovascular Accident, Acute)) OR TS=(Acute Strokes)) OR TS=(Acute Stroke)) OR TS=(Acute Cerebrovascular Accidents)) OR TS=(Acute Cerebrovascular Accident)) OR TS=(Stroke, Acute)) OR TS=(Strokes, Acute)<br>#4 #3 AND #2 AND #1
Cochrane Library	#1 MeSH descriptor: [Music Therapy] explode all trees<br>#2 (Therapy, Music):ti,ab,kw OR (music):ti,ab,kw<br>#3 #1 OR #2<br>#4 MeSH descriptor: [Sleep Wake Disorders] explode all trees<br>#5 (Sleeper Syndrome, Long OR Sleeper Syndromes,Long OR Long Sleeper Syndrome OR Syndromes, Long Sleeper OR Long Sleeper Syndromes OR Neurogenic Tachypnea, Sleep-Related OR Sleep Related Neurogenic Tachypnea OR achypneas, Sleep-Related Neurogenic OR Sleep-Related Neurogenic Tachypnea OR Short Sleeper Syndrome OR Short Sleeper Syndromes OR Phenotype, Short Sleep OR Sleeper Syndromes, Short OR Short Sleeper OR Short Sleep Phenotype OR Subwakefullness Syndrome OR Syndromes, Subwakefullness OR Subwakefullness Syndromes OR Syndrome, Subwakefullness OR Disorders, Sleep Wake OR Wake Disorder, Sleep OR Wake Disorders, Sleep OR Sleep Wake Disorder OR Disorder, Sleep Wake OR Sleep Disorders OR Disorders, Sleep OR Disorder, Sleep OR Sleep Disorder):ti,ab,kw<br>#6 #4 OR #5<br>#7 MeSH descriptor: [Stroke] explode all trees<br>#8 (Strokes OR CVA (Cerebrovascular Accident) OR Apoplexy OR Cerebrovascular Accident OR Cerebrovascular Stroke OR Vascular Accident, Brain OR Brain Vascular Accidents OR Cerebrovascular Strokes OR Stroke, Cerebrovascular OR Cerebral Strokes OR Vascular Accidents, Brain OR Strokes, Cerebral OR Cerebrovascular Accidents OR Strokes, Cerebrovascular OR Brain Vascular Accident OR Cerebral Stroke OR Cerebrovascular Apoplexy OR Stroke, Cerebral OR Apoplexy, Cerebrovascular OR CVAs (Cerebrovascular Accident) OR Cerebrovascular Accidents, Acute OR Cerebrovascular Accident, Acute OR Acute Strokes OR Acute Stroke OR Acute Cerebrovascular Accidents OR Acute Cerebrovascular Accident OR Stroke, Acute OR Strokes, Acute):ti,ab,kw<br>#9 #7 OR #8<br>#10 #3 AND #6 AND #9
Pubmed	#1 ((music therapy[MeSH Terms]) OR (Therapy, Music[Title/Abstract])) OR (music[Title/Abstract])<br>#2 ((((((((((((((((((((((((((\"Dyssomnias\"[Mesh]) OR (Dyssomnia[Title/Abstract])) OR (Sleep Disorders, Extrinsic[Title/Abstract])) OR (Extrinsic Sleep Disorder[Title/Abstract])) OR (Extrinsic Sleep Disorders[Title/Abstract])) OR (Sleep Disorder, Extrinsic[Title/Abstract])) OR (Adjustment Sleep Disorder[Title/Abstract])) OR (Adjustment Sleep Disorders[Title/Abstract])) OR (Sleep Disorders, Adjustment[Title/Abstract])) OR (Sleep Disorder, Adjustment[Title/Abstract])) OR (Environmental Sleep Disorder[Title/Abstract])) OR (Environmental Sleep Disorders[Title/Abstract])) OR (Sleep Disorders, Environmental[Title/Abstract])) OR (Sleep Disorder, Environmental[Title/Abstract])) OR (Limit-Setting Sleep Disorder[Title/Abstract])) OR (Limit Setting Sleep Disorder[Title/Abstract])) OR (Limit-Setting Sleep Disorders[Title/Abstract])) OR (Sleep Disorders, Limit-Setting[Title/Abstract])) OR (Sleep Disorder, Limit-Setting[Title/Abstract])) OR (Sleep Disorder, Limit Setting[Title/Abstract])) OR (Nocturnal Eating-Drinking Syndrome[Title/Abstract])) OR (Eating-Drinking Syndrome, Nocturnal[Title/Abstract])) OR (Eating-Drinking Syndromes, Nocturnal[Title/Abstract])) OR (Nocturnal Eating Drinking Syndrome[Title/Abstract])) OR (Nocturnal Eating-Drinking Syndromes[Title/Abstract])) OR (Syndrome, Nocturnal Eating-Drinking[Title/Abstract])) OR (Syndromes, Nocturnal Eating-Drinking[Title/Abstract])

	#3 (((((((((((((((((((((((((("Stroke"[Mesh]) OR (Strokes[Title/Abstract])) OR (Cerebrovascular Accident[Title/Abstract])) OR (Cerebrovascular Accidents[Title/Abstract])) OR (Cerebral Stroke[Title/Abstract])) OR (Cerebral Strokes[Title/Abstract])) OR (Stroke, Cerebral[Title/Abstract])) OR (Strokes, Cerebral[Title/Abstract])) OR (Cerebrovascular Apoplexy[Title/Abstract])) OR (Apoplexy, Cerebrovascular[Title/Abstract])) OR (Vascular Accident, Brain[Title/Abstract])) OR (Brain Vascular Accident[Title/Abstract])) OR (Brain Vascular Accidents[Title/Abstract])) OR (Vascular Accidents, Brain[Title/Abstract])) OR (Cerebrovascular Stroke[Title/Abstract])) OR (Cerebrovascular Strokes[Title/Abstract])) OR (Stroke, Cerebrovascular[Title/Abstract])) OR (Strokes, Cerebrovascular[Title/Abstract])) OR (Apoplexy[Title/Abstract])) OR (CVA (Cerebrovascular Accident[Title/Abstract]))) OR (CVAs (Cerebrovascular Accident[Title/Abstract]))) OR (Stroke, Acute[Title/Abstract])) OR (Acute Stroke[Title/Abstract])) OR (Acute Strokes[Title/Abstract])) OR (Strokes, Acute[Title/Abstract])) OR (Cerebrovascular Accident, Acute[Title/Abstract])) OR (Acute Cerebrovascular Accident[Title/Abstract])) OR (Acute Cerebrovascular Accidents[Title/Abstract])) OR (Cerebrovascular Accidents, Acute[Title/Abstract])<br>#4 #1 and #2 and #3
CNKI	（主题：音乐疗法 + 音乐疗法治疗 + 音乐疗法干预 + 音乐疗法曲目 +'音乐疗法/方法'+ 五行音乐疗法 + 中医五行音乐疗法 + 中医音乐疗法 + 接受式音乐疗法 + 主动音乐疗法)AND(主题:卒中后睡眠障碍 + 中风后睡眠障碍 + 卒中后失眠 + 卒中后不寐 + 中风后失眠 + 中风后不寐 + 脑卒中 + 中风 + 失眠 + 睡眠障碍 + 不寐）AND（篇关摘：随机对照 + 随机对照试验 + 随机对照研究 + 随机对照临床试验 + 随机对照方法 + 随机对照临床研究(模糊)）
vip	((((((((题名或关键词=音乐干预 OR 题名或关键词=音乐治疗) OR 题名或关键词=音乐疗法) OR 题名或关键词=五行音乐) OR 题名或关键词=中医音乐) OR 题名或关键词=中医五行音乐) OR 题名或关键词=音乐) AND (((((((题名或关键词=睡眠障碍 OR 题名或关键词=失眠) OR 题名或关键词=不寐) OR 题名或关键词=卒中后睡眠障碍) OR 题名或关键词=中风后睡眠障碍) OR 题名或关键词=脑卒中) OR 题名或关键词=卒中) OR 题名或关键词=中风)) AND ((((摘要=临床研究 OR 摘要=临床试验) OR 摘要=随机对照) OR 摘要=随机) OR 摘要=随机分组))
Wanfang Data	检索表达式（中英文扩展&主题词扩展）：题名或关键词:(音乐治疗 OR 音乐 OR 音乐疗法 OR 五行音乐治疗 OR 五行音乐疗法 OR 中医音乐治疗 OR 中医音乐疗法) and 题名或关键词:(睡眠障碍 OR 失眠 OR 不寐 OR 脑卒中 OR 卒中 OR 中风 OR 卒中后睡眠障碍 OR 中风后睡眠障碍) and 摘要:(随机对照 OR 随机分组 OR 随机 OR 临床试验 OR 临床疗效 OR 临床)
