# Supplementary material for: Effects of music on post-stroke sleep disorders and treatment perspectives: review and narrative synthesis
Source: Front Hum Neurosci. 2026 Jan 14;19:1710535. doi: 10.3389/fnhum.2025.1710535 (PMC12847286; doi:10.3389/fnhum.2025.1710535)
Supplement: Supplementary file 2 [file Data_Sheet_2.pdf]

Appendix b Inclusion of 14 studies

REFERENCE:

(1-14)
1.Ma, T. 2016. "Effect of music therapy on sleep disorders in patients after ischemic stroke." Chinese Journal of Urban and Rural Enterprise Hygiene 31(4):68-69.
2.Jia, X.X. 2021. "Observation on the effect of Wuyin therapy in auxiliary treatment of post-stroke insomnia." China Practical Medicine 43(6):1623-1626.
3.Chang, M.N., Y.Y. Wang, and H. Zhang. 2020. "Application effect of five tone characteristic nursing intervention in patients with sleep disorders of stroke." Clinical Research and Practice 5(15):141-142.
4.Yang H.Y., Feng X.G., Hao W.J., Wang Y., LI J., AND Wu X.L., 2016 "The application of traditional Chinese medicine music therapy in post- stroke depression patients" Nursing Practice and Research 13(14):134-136.
5.Zhang M.Y., Li L., Ding Y., 2025. "Effects of traditional Chinese medicine five elements music and western classical music on sleep quality of stroke patients". Chinese Journal of Practical Nursing 41(6):428-436
6.Duan Yi;LUO Feng;LIU Ling. 2018."Effect of Group Mode Music Therapy on Sleep Quality of Patients with Post-stroke Depression." Clinical Research 26(5):17-18.
7.Li, F.X. 2020. "Effect of Music Nursing Combined with Estazolam on Sleep Disorders in Stroke Patients." World Journal of Sleep Medicine 4:691-692.
8.Li, G.Y., Y.T. Du, Y. Yang, and X.M. Fu. 2022. "Observation of the effect of high-low frequency conversion hearing-exercise training on elderly patients with cerebral hemorrhage during sleep." Jilin Medical Journal 6:1623-1626.
9.Wang, Q., J.Z. Wang, and J. Cheng. 2019. "Effect of high-low frequency conversion hearing-exercise training on sleep in patients with cerebral hemorrhage." Chinese Nursing Management 19(10):1491-1496.
10.Huang, Z.Y., and F.P. Deng. 2019. "Application effect of small-dose doxepin combined with five elements music therapy in patients with post-stroke sleep disorder." Journal of Changzhi Medical College 1:65-67.
11.Li Xuelian;Tang Linfang;Deng Xu;Zeng Qing;LI Li;Liamg Shuping 2025 "Efficacy of five elements music therapy combined with Leihuo moxibustion on insomnia patients with heart-spleen deficiency type after ischemic stroke". Journal of Guangxi University Of Chinese Medicine 28(3):40-43.
12.Zhang Y., Li L.X.,Famg L.Q.,Hao D.X., AND Zhamg X.P., 2016 "Curative effect of five elements music combined with auricular point sticking on post-s troke insomnia by PSQI". China Journal of Traditional Chinese Medicine and Pharmacy 31(08):3063-3065.
13.Wang Y., Ding Z S., 2013 "Efficacy Observation of Post Stroke Insomnia Treated with Acupuncture and Music Therapy" World Journal of Integrated Traditional and Western Medicine 8(11):1136-1138.DOI:10.13935/j.cnki.sjzx.2013.11.012.
14.Cai XM, Zhang XP, Tang X. 2015 "Observation on clinical effect of auricular acupoint sticking plus music therapy for post-stroke insomnia." J Acupunct Tuina Sci,

13 (4): 227-231DOI: 10.1007/s11726-015-0858-2
