# Supplementary material for: Effects of music on post-stroke sleep disorders and treatment perspectives: review and narrative synthesis
Source: Front Hum Neurosci. 2026 Jan 14;19:1710535. doi: 10.3389/fnhum.2025.1710535 (PMC12847286; doi:10.3389/fnhum.2025.1710535)
Supplement: Supplementary file 3 [file Data_Sheet_3.pdf]

Appendix c Table of results for each study								
study	***treatment***	***mean***	***SD***	***MD***	***95% CI***	***p-values***	***outcomes***	***sample size***
Ma 2016	*TCFM*	*6.03*	*1.2*	*-1.55*	*(-2.1219, -0.9781)*	*p-value<0.05*	*PSQI*	*38*
Ma 2016	*Routine treatment*	*7.58*	*1.3*				*PSQI*	*38*
Jia 2021	*FMT*	*6.84*	*2.11*	*-1.36*	*(-2.5281, -0.2119)*	*p-value<0.05*	*PSQI*	*25*
Jia 2021	*Estazolam*	*8.21*	*1.96*				*PSQI*	*25*
Chang 2020	*FMT*	*11.23*	*2.52*	*-3.94*	*(-4.8553, -3.0247)*	*p-value<0.01*	*PSQI*	*73*
Chang 2020	*Routine treatment*	*15.17*	*3.05*				*PSQI*	*73*
HY YANG 2016	*FMT*	*8.48*	*3.46*	*-0.71*	*(-1.9606, 0.5406)*	*p-value<0.05*	*PSQI*	*69*
YANG 2016	*Fluoxetine hydrochloride*	*9.19*	*3.93*				*PSQI*	*68*
ZHANG 2025	*FMT*	*7.64*	*2.08*	*-4.58*	*(-6.0503, -3.1097)*	*p-value<0.001*	*PSQI*	*25*
ZHANG 2025	*Routine treatment*	*12.22*	*2.94*				*PSQI*	*23*
ZHANG 2025	*WCM*	*10.33*	*2.82*	*-2.19*	*(-3.5822, -0.1978)*	*p-value<0.001*	*PSQI*	*24*
ZHANG 2025	*Routine treatment*	*12.22*	*2.94*				*PSQI*	*23*
Duan 2018	*GMMT*	*5.2*	*1.2*	*-4.1*	*(-4.8341, -3.3659)*	*p-value<0.001*	*PSQI*	*46*
Duan 2018	*Routine treatment+Sedative music*	*9.3*	*2.2*				*PSQI*	*46*
LI 2020	*MT+Estazolam*	*5.81*	*1.24*	*-2.14*	*(-2.9031, -1.3769)*	*p-value<0.001*	*PSQI*	*30*
LI 2020	*Estazolam*	*7.95*	*1.68*				*PSQI*	*30*
LI 2022	*High —low frequency conversion training*	*2.19*	*0.49*	*-0.08*	*(-0.2656, 0.1056)*	*p-value=0.395*	*Sleep Latency*	*57*
LI 2022	*Routine treatment*	*2.27*	*0.51*				*Sleep Latency*	*57*
LI 2022	*High —low frequency*	*2.42*	*0.51*	*-0.05*	*(-0.2393, 0.1393)*	*p-value=0.623*	*Sleep Duration*	*57*

	conversion training							
LI 2022	Routine treatment	2.47	0.51				Sleep Duration	57
LI 2022	High —low frequency conversion training	1.97	0.44	-0.34	(-0.5071, -0.1729)	p-value<0.001	Subjective Sleep Quality	57
LI 2022	Routine treatment	2.31	0.46				Subjective Sleep Quality	57
I 2022	High —low frequency conversion training	2.20	0.51	-0.21	(-0.3956, -0.0244)	p-value=0.027	Habitual Sleep Efficiency	57
LI 2022	Routine treatment	2.41	0.49				Habitual Sleep Efficiency	57
LI 2022	High —low frequency conversion training	4.16	0.98	-3.42	(-3.7893, -3.0507)	p-value<0.001	Sleep Disturbances	57
LI 2022	Routine treatment	7.58	1.01				Sleep Disturbances	57
LI 2022	High —low frequency conversion training	1.08	0.40	-0.04	(-0.1903, 0.1103)	p-value=0.559	Use of Sleep Medications	57
LI 2022	Routine treatment	1.12	0.41				Use of Sleep Medications	57
LI 2022	High —low frequency conversion training	4.48	0.63	-0.04	(-0.2833, 0.2033)	p-value=0.257	Daytime Dysfunction	57
LI 2022	Routine treatment	4.52	0.68				Daytime Dysfunction	57
WANG 2019	High —low frequency conversion training	1.95	0.79	-0.03	(-0.3478, 0.2878)	p-value=0.024	Subjective Sleep Quality	42
WANG 2019	Mozart's Music+Gregorian Chant	1.98	0.68				Subjective Sleep Quality	43

WANG 2019	*High —low frequency conversion training*	*2.19*	*0.74*	*0*	*(-0.3193, 0.3193)*	*p-value=0.990*	*Sleep Latency*	*42*
WANG 2019	*Mozart's Music+Gregorian Chant*	*2.19*	*0.74*				*Sleep Latency*	*43*
WANG 2019	*High —low frequency conversion training*	*2.50*	*0.67*	*0.19*	*(-0.124, 0.504)*	*p-value=0.466*	*Sleep Duration*	*42*
WANG 2019	*Mozart's Music+Gregorian Chant*	*2.31*	*0.78*				*Sleep Duration*	*43*
WANG 2019	*High —low frequency conversion training*	*2.38*	*0.69*	*0.24*	*(-0.0711, 0.5511)*	*p-value=0.304*	*Habitual Sleep Efficiency*	*42*
WANG 2019	*Mozart's Music+Gregorian Chant*	*2.14*	*0.75*				*Habitual Sleep Efficiency*	*43*
HUANG 2019	*FMT+Doxepin hydrochloride*	*7.58*	*1.67*	*-1.95*	*(-2.5653, -1.3347)*	*p-value=0.008*	*PSQI*	*57*
HUANG 2019	*Doxepin hydrochloride*	*9.53*	*1.63*				*PSQI*	*56*
Li 2025	*FMT+Leihuo moxibustion*	*8.69*	*2.01*	*-3.17*	*(-4.2599, -2.0801)*	*p-value<0.05*	*PSQI*	*35*
Li 2025	*Leihuo moxibustion*	*11.86*	*2.53*				*PSQI*	*35*
ZHANG 2016	*FMT+Auricular acupoint sticking*	*8.56*	*5.84*	*-3.27*	*(-5.4471, -1.0929)*	*p-value<0.05*	*PSQI*	*52*
ZHANG 2016	*Auricular acupoint sticking*	*11.83*	*5.21*				*PSQI*	*50*
Chang 2020	*FMT*	*0.97*	*0.11*	*0.13*	*(0.099 , 0.161)*	*p-value<0.01*	*sleep efficiency*	*73*
Chang 2020	*Routine treatment*	*0.84*	*0.12*				*sleep efficiency*	*73*
Chang 2020	*FMT*	*42.16*	*3.11*	*-1.21*	*(-2.33 , -0.09)*	*p-value<0.01*	*sleep latency*	*73*
Chang 2020	*Routine treatment*	*43.37*	*3.03*				*sleep latency*	*73*
Chang 2020	*FMT*	*1.97*	*0.21*	*-0.86*	*(–1.17 , –0.55)*	*p-value<0.01*	*number of awakenings*	*73*
Chang	*Routine treatment*	*2.83*	*0.92*				*number of*	*73*

2020							awakenings	
Chang 2020	FMT	25.57	3.15	-2.76	(-3.83 , -1.69)	p-value<0.01	SRSS	73
Chang 2020	Routine treatment	28.33	3.41				SRSS	73
ZHANG 2025	FMT	6.76	1.36	-2.72	(-3.54 , -1.90)	p-value<0.001	AIS	25
ZHANG 2025	Routine treatment	9.48	1.53				AIS	23
ZHANG 2025	WCM	7.46	1.38	-2.02	(-2.86 , -1.18)	p-value<0.001	AIS	24
ZHANG 2025	Routine treatment	9.48	1.53				AIS	23
WANG 2013	Sedative music+Acupuncture	10.22	1.56	-1.56	(-2.26 , -0.86)	p-value<0.05	SSI	46
WANG 2013	Acupuncture	11.78	1.84				SSI	46
Chang 2020	FMT	0.97	0.11	0.13	(0.099 , 0.161)	p-value<0.01	sleep efficiency	73
Chang 2020	Routine treatment	0.84	0.12				sleep efficiency	73
Chang 2020	FMT	42.16	3.11	-1.21	(-2.33 , -0.09)	p-value<0.01	sleep latency	73
Chang 2020	Routine treatment	43.37	3.03				sleep latency	73
Chang 2020	FMT	1.97	0.21	-0.86	(–1.17 , –0.55)	p-value<0.01	number of awakenings	73
Chang 2020	Routine treatment	2.83	0.92				number of awakenings	73
ZHANG 2025	FMT	399.89	51.76	54.41	(28.68, 80.13)	p-value<0.05	Total Sleep Time	25
ZHANG 2025	Routine treatment	345.48	38.69				Total Sleep Time	23
ZHANG 2025	WCM	368.45	47.88	22.97	(-1.87, 47.81)	p-value >0.05	Total Sleep Time	24
ZHANG 2025	Routine treatment	345.48	38.69				Total Sleep Time	23
ZHANG 2025	FMT	28.86	17.68	-13.52	(-23.91, -3.12)	p-value<0.05	sleep latency	25
ZHANG 2025	Routine treatment	42.38	18.96				sleep latency	23
ZHANG 2025	WCM	35.67	16.99	-6.71	(-17.02, 3.60)	p-value >0.05	sleep latency	24

ZHANG 2025	Routine treatment	42.38	18.96				sleep latency	23
ZHANG 2025	FMT	78.38	37.26	24.01	(6.15, 41.87)	p-value<0.05	Wake After Sleep Onset	25
ZHANG 2025	Routine treatment	54.37	25.15				Wake After Sleep Onset	23
ZHANG 2025	WCM	64.28	29.34	9.91	(-5.69, 25.51)	p-value >0.05	Wake After Sleep Onset	24
ZHANG 2025	Routine treatment	54.37	25.15				Wake After Sleep Onset	23
ZHANG 2025	FMT	2.72	1.19	-2.15	(-2.91, -1.39)	p-value<0.05	number of awakenings	25
ZHANG 2025	Routine treatment	4.87	1.46				number of awakenings	23
ZHANG 2025	WCM	3.67	1.12	-1.2	(-1.95, -0.45)	p-value<0.05	number of awakenings	24
ZHANG 2025	Routine treatment	4.87	1.46				number of awakenings	23
ZHANG 2025	FMT	136.26	25.36	24.89	(11.14, 38.64)	p-value<0.05	REM Sleep Latency	25
ZHANG 2025	Routine treatment	111.37	23.23				REM Sleep Latency	23
ZHANG 2025	WCM	124.46	26.25	13.09	(-1.07, 27.25)	p-value >0.05	REM Sleep Latency	24
ZHANG 2025	Routine treatment	111.37	23.23				REM Sleep Latency	23
ZHANG 2025	FMT	78.87	8.21	9.43	(6.10, 12.76)	p-value<0.05	sleep efficiency	25
ZHANG 2025	Routine treatment	69.44	7.88				sleep efficiency	23
ZHANG 2025	WCM	73.36	7.86	3.92	(0.50, 7.34)	p-value >0.05	sleep efficiency	24
ZHANG 2025	Routine treatment	69.44	7.88				sleep efficiency	23
ZHANG 2025	FMT	95.71	15.23	14.78	(5.78, 23.78)	p-value<0.05	Sleep Time(N1)	25
ZHANG 2025	Routine treatment	80.93	16.47				Sleep Time(N1)	23
ZHANG 2025	WCM	83.2	18.34	2.27	(-7.68, 12.23)	Not mentioned	Sleep Time(N1)	24
ZHANG 2025	Routine treatment	80.93	16.47				Sleep Time(N1)	23
ZHANG 2025	FMT	21.87	7.89	-0.3	(-4.48, 3.88)	Not mentioned	Sleep Stage Proportions	25

							(N1)	
ZHANG 2025	Routine treatment	22.17	6.87				Sleep Stage Proportions (N1)	23
ZHANG 2025	WCM	20.36	8.35	-1.81	(-6.17, 2.55)	Not mentioned	Sleep Stage Proportions (N1)	24
ZHANG 2025	Routine treatment	22.17	6.87				Sleep Stage Proportions (N1)	23
ZHANG 2025	FMT	156.67	40.16	12.39	(-9.92, 34.70)	Not mentioned	Sleep Time(N2)	25
ZHANG 2025	Routine treatment	144.28	38.67				Sleep Time(N2)	23
ZHANG 2025	WCM	150.97	34.33	6.69	(-14.25, 27.63)	Not mentioned	Sleep Time(N2)	24
ZHANG 2025	Routine treatment	144.28	38.67				Sleep Time(N2)	23
ZHANG 2025	FMT	34.58	7.98	-5.31	(-10.10, -0.52)	Not mentioned	Sleep Stage Proportions (N2)	25
ZHANG 2025	Routine treatment	39.89	8.88				Sleep Stage Proportions (N2)	23
ZHANG 2025	WCM	39.56	8.82	-0.33	(-5.39, 4.73)	Not mentioned	Sleep Stage Proportions (N2)	24
ZHANG 2025	Routine treatment	39.89	8.88				Sleep Stage Proportions (N2)	23
ZHANG 2025	FMT	84.23	20.98	38.36	(27.15, 49.57)	p-value<0.05	Sleep Time(N3)	25
ZHANG 2025	Routine treatment	45.87	18.65				Sleep Time(N3)	23
ZHANG 2025	WCM	65.33	18.82	19.46	(8.75, 30.17)	p-value<0.05	Sleep Time(N3)	24
ZHANG 2025	Routine treatment	45.87	18.65				Sleep Time(N3)	23
ZHANG 2025	FMT	23.98	5.89	8.95	(5.71, 12.19)	p-value<0.05	Sleep Stage Proportions (N3)	25
ZHANG 2025	Routine treatment	15.03	5.56				Sleep Stage Proportions (N3)	23

ZHANG 2025	*WCM*	*18.34*	*4.78*	*3.31*	*(0.58, 6.04)*	*p-value<0.05*	*Sleep Stage Proportions (N3)*	*24*
ZHANG 2025	*Routine treatment*	*15.03*	*5.56*				*Sleep Stage Proportions (N3)*	*23*
ZHANG 2025	*FMT*	*85.34*	*17.35*	*6.46*	*(-4.61, 17.53)*	*Not mentioned*	*Sleep Time(REM)*	*25*
ZHANG 2025	*Routine treatment*	*78.88*	*21.36*				*Sleep Time(REM)*	*23*
ZHANG 2025	*WCM*	*86.35*	*19.99*	*7.47*	*(-4.37, 19.31)*	*Not mentioned*	*Sleep Time(REM)*	*24*
ZHANG 2025	*Routine treatment*	*78.88*	*21.36*				*Sleep Time(REM)*	*23*
ZHANG 2025	*FMT*	*24.78*	*6.67*	*2.99*	*(-0.72, 6.70)*	*Not mentioned*	*Sleep Stage Proportions (REM)*	*25*
ZHANG 2025	*Routine treatment*	*21.79*	*6.54*				*Sleep Stage Proportions (REM)*	*23*
ZHANG 2025	*WCM*	*23.56*	*6.78*	*1.77*	*(-2.01, 5.55)*	*Not mentioned*	*Sleep Stage Proportions (REM)*	*24*
ZHANG 2025	*Routine treatment*	*21.79*	*6.54*				*Sleep Stage Proportions (REM)*	*23*

study	***treatment***	***sample***	***rate***	***RD***	***95% confidence***	***p-values***	***outcomes***	***sample size***
Jia 2021	*FMT*	*24*	*96.00%*	*0.94*	*(0.0158, 0.3842)*	*p-value<0.05*	*ORR*	*25*
Jia 2021	*Estazolam*	*19*	*76.00%*				*ORR*	*25*
LI 2020	*MT+Estazolam*	*29*	*96.67%*	*0.09*	*(0.0626, 0.4042)*	*p-value=0.030*	*ORR*	*30*
LI 2020	*Estazolam*	*22*	*73.33%*				*ORR*	*30*
ZHANG 2016	*FMT+Auricular acupoint sticking*	*46*	*88.50%*	*0.07*	*(-0.0562, 0.2254)*	*p-value<0.05*	*ORR*	*52*
ZHANG 2016	*Auricular acupoint sticking*	*40*	*80.00%*				*ORR*	*50*

Jia 2021	*FMT*	*0*	*0%*	*0.09*	*(0.0052, 1.598)*	*p-value<0.05*	*AER*	*25*
Jia 2021	*Estazolam*	*5*	*20%*				*AER*	*25*
